# Advances in Understanding the Roles of CD244 (SLAMF4) in Immune Regulation and Associated Diseases

**DOI:** 10.3389/fimmu.2021.648182

**Published:** 2021-03-24

**Authors:** Lin Sun, Xiaokun Gang, Zhuo Li, Xue Zhao, Tong Zhou, Siwen Zhang, Guixia Wang

**Affiliations:** Department of Endocrinology and Metabolism, The First Hospital of Jilin University, Changchun, China

**Keywords:** SLAMF4, disease models, autoimmune disease, immune cells, immune regulation

## Abstract

Proteins in the signaling lymphocytic activating molecule (SLAM) family play crucial roles in regulating the immune system. CD244 (SLAMF4) is a protein in this family, and is also a member of the CD2 subset of the immunoglobulin (Ig) superfamily. CD244 is a cell surface protein expressed by NK cells, T cells, monocytes, eosinophils, myeloid-derived suppressor cells, and dendritic cells. CD244 binds to the ligand CD48 on adjacent cells and transmits stimulatory or inhibitory signals that regulate immune function. In-depth studies reported that CD244 functions in many immune-related diseases, such as autoimmune diseases, infectious diseases, and cancers, and its action is essential for the onset and progression of these diseases. The discovery of these essential roles of CD244 suggests it has potential as a prognostic indicator or therapeutic target. This review describes the molecular structure and function of CD244 and its roles in various immune cells and immune-related diseases.

## Introduction

CD244 (SLAMF4) is a transmembrane protein present in NK cells, T cells, and other types of immune cells. This protein is a key cell surface receptor, a member of the signaling lymphocytic activating molecule (SLAM) family, and functions as a receptor in immune regulation (1, 2). CD244 mainly binds to the CD48 (another member of the SLAM family) on other immune cells, thus regulating the immune response by a *trans* interaction (3–5). In addition to the trans interaction, there is also cis interaction between CD244 and CD48 in NK cells, 2B4/CD48 interaction is essential for the expansion and activation of murine NK cells. In the absence of 2B4/CD48 interaction, NK cytotoxicity and IFN-γ secretion on tumor target exposure is severely impaired (6, 7). Although CD244 can also bind to other low-affinity ligands such as CD229, most studies have focused on the interaction of CD244 and CD48 (4, 8) and on the intracellular SLAM-associated protein (SAP) via its immunoreceptor tyrosine-based switch motifs (ITSMs) (4, 9). CD244 provides stimulatory or inhibitory signals that regulate various immune responses in NK cells, CD8^+^ T cells, and other immune cells, such as cytotoxicity, cytokine production, and intercellular interactions. The cellular and molecular effects of CD244 suggest it may function in the onset and progression of multiple immune-related diseases.

Abnormal interactions between immune cells contribute to the onset and progression of autoimmune diseases, such as systemic lupus erythematosus (SLE), rheumatoid arthritis (RA), and type 1 diabetes (T1D) (10–12). Co-signaling molecules, such as CD244/CD48, affect the onset of autoimmune diseases by regulating various functions of immune cells and by maintaining or altering the balance of immune responses (13). Infectious diseases also cause dramatic changes in the immune system that promote the clearing of foreign antigens. In antiviral immunity, the normal functions of virus-specific CD8^+^ T cells and NK cells depend on CD244 expression and transmission of appropriate stimulatory or inhibitory signals (14). However, there is evidence that patients with active tuberculosis (TB) achieve immune suppression due to increased expression of CD244 by many immune cells, including CD4^+^ T cells and myeloid-derived suppressor cells (MDSCs) (15). The increased expression of CD244 and its ligands by various immune cells can stimulate immune cell function, but can also contribute to sepsis by disturbing systemic immune function. A recent study characterized sepsis as systemic life-threatening dysfunction of organs caused by a dysregulated host response to infection, and thus a systemic immune disorder (16).

Tumors are another challenge to the immune system. Various immune cells, fibroblasts, other types of cells, extracellular components, and the surrounding vascular network system constitute the microenvironment of tumors, and these all affect tumor development and metastasis (17–19). Whether the different immune cells in the tumor microenvironment promote removal of the tumor or immune escape determines the outcome and prognosis of the tumor. CD244 is on the surfaces of various immune cells in the tumor microenvironment, and it helps to regulate immune function, and plays an essential role in tumor onset and development. Moreover, CD244 can be used as a diagnostic and prognostic marker, and may be useful for the immunotherapeutic treatment of tumors (20). This review describes the molecular structural similarities and differences of CD244 and other proteins in the SLAM family, summarizes the established roles of CD244 in various diseases, and provides a theoretical basis for the future use of CD244 in the diagnosis and treatment of several specific diseases. This review also describes the possible immunological roles of CD244 in additional diseases, and the prospects for using CD244 in the diagnosis and treatment of these other diseases.

## Molecular Characteristics of CD244

### Structure

**Figure 1:**
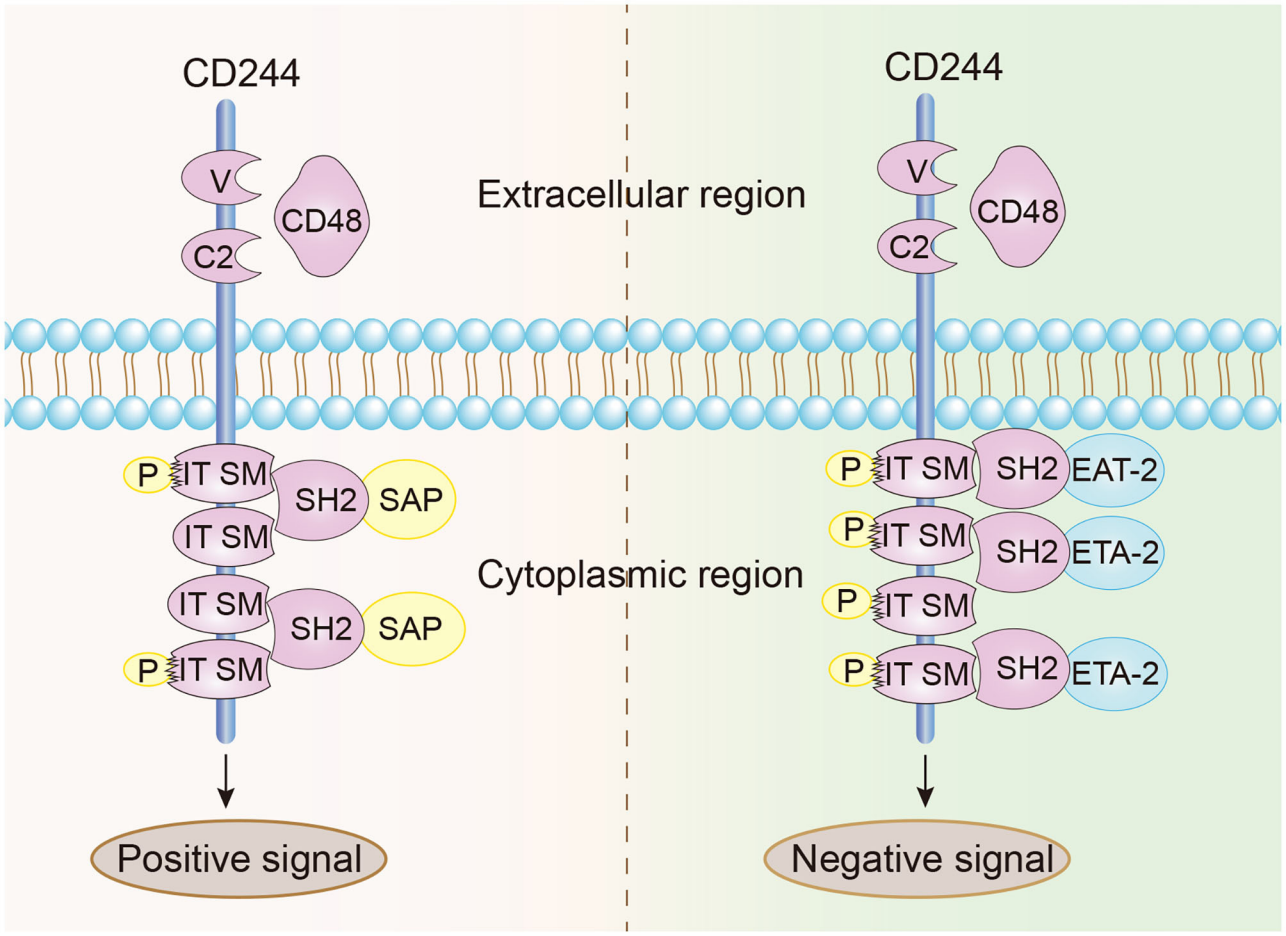
Structural characteristics and signal transmission model of CD244. CD244 is a transmembrane receptor that binds with high affinity to extracellular CD48. Positive intracellular signal transduction depends on the binding of ITSMs with SAP, and negative intracellular signal transduction depends on the binding of ITSMs with ERT and EAT2.

CD244 is a type-1 transmembrane protein consisting of an N-terminal variable Ig domain with two constant Ig domains, a structural feature of most proteins in the CD2 family that contain the Ig domain (21). The cytoplasmic domain of CD244 has 4 immunoreceptor tyrosine-based switch motifs (ITSMs), denoted as TxYxxI/V (where “x” indicates any amino acid), and these motifs occur in all proteins in the SLAM family. These motifs are needed for binding to SAP, which contains the Src homology 2 (SH2) domain and is responsible for the transmission of stimulatory and inhibitory signals (Figure 1) (22, 23). Notably, ITSMs can only bind with SAP and other protein molecules after the tyrosine residues are phosphorylated (24–26). Unlike other SLAM family receptors, CD244 is the only heterophilic receptor that binds to the glycosyl phosphodyl inositol (GPI) -anchored ligand CD48 for signal transmission; all other receptors in this family are homophilic receptors and function as self-ligands that transmit signals (27–30). In addition to SAP, CD244 also binds to Ewing sarcoma-activated transcript 2 (EAT2) and several phosphatases (SHP1, SHP2, and SHI-1), and transmits activation or inhibition signals that lead to different immunomodulatory effects (31–33).

### Function

Researchers first discovered CD244 in NK cells and CD8^+^ T cells, and initially characterized it as a stimulatory cell surface receptor that mediated non-MHC-restricted killing by NK cells and CD8^+^ T cells. NK cells and CD8^+^ T cells have numerous surface receptors, and can attack antibody-coated target cells and release cytokines (34, 35). Previous research indicated that CD244 activated NK cell-mediated cytotoxicity and induced the secretion of IFN-γ by activating the AP-1 pathway and the RAS-dependent mitogen activated-protein kinase (MAPK) pathway (23, 36). Blockage of CD244 and CD48 binding also reduced the cytotoxicity of CD8^+^ T cells and the expression of CD8^+^ T cell functional effectors (IFN-γ, TNF, MIP-1β, perforin, and granzyme B) (22).

**Figure 2:**
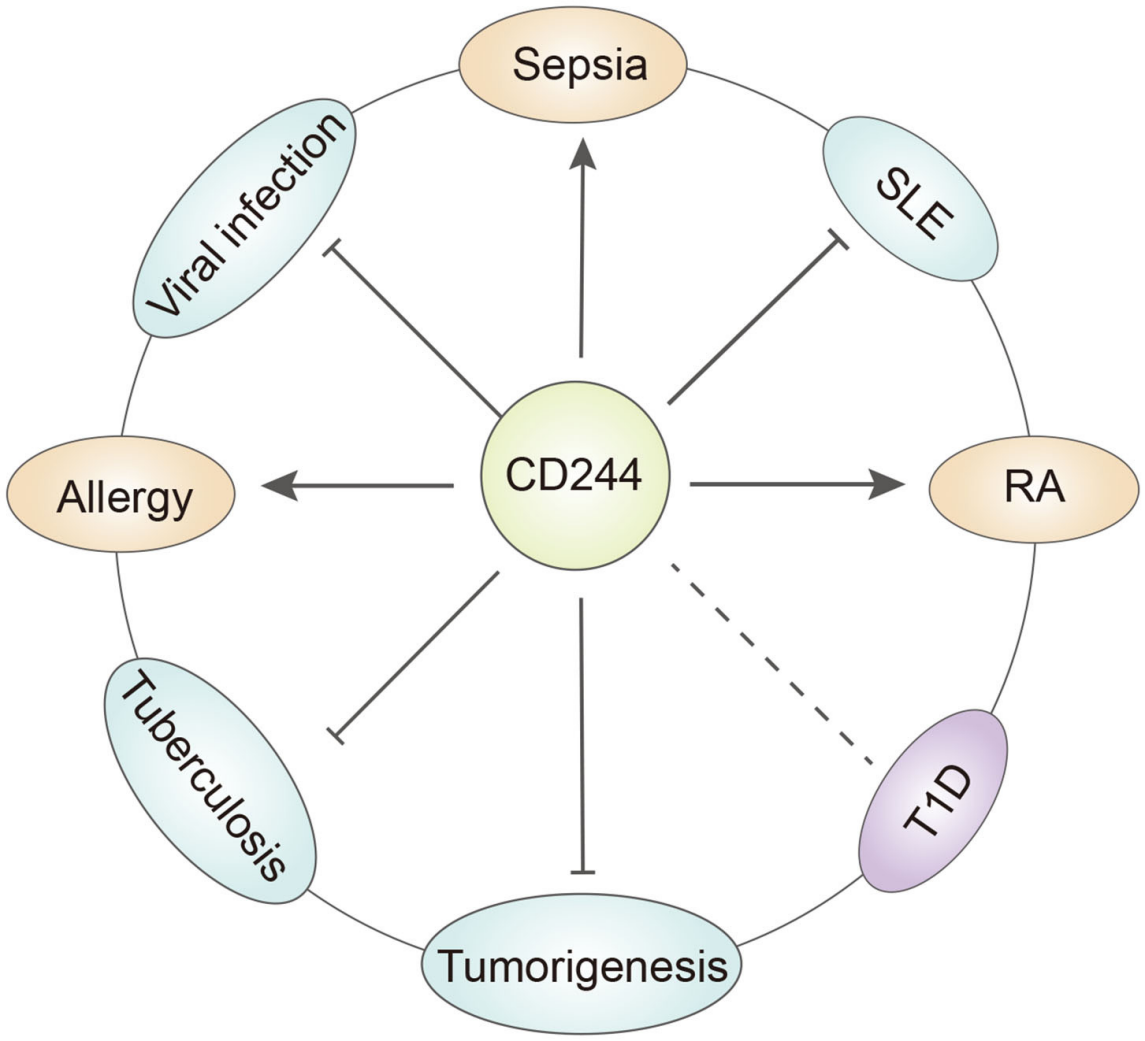
The role of CD244 in different diseases. CD244 has quite different function under specific disease condition. T1D, type a diabetes. SLE, systemic lupus erythematosus. RA, rheumatoid arthritis.

However, recent studies of CD244 function reported opposite results. In particular, McNerney et al. suggested that CD244-deficient NK cells were more cytotoxic than wild-type NK cells (21). Moreover, addition of CD244 to wild-type NK cells inhibited cell toxicity and IFN production, and the same inhibitory effect occurred in memory CD8^+^ T cells. In chronic infections, CD244 limited the recall response of CD8^+^ T cells by inhibiting their proliferation and function (37). The reason for these different results may be that CD244 binds to multiple protein receptors that transmit different intracellular signals. For instance, the binding of CD244 to SAP upregulates the viability and cytotoxic effects of NK cells and CD8^+^ T cells, but its binding to EAT2 may transmit inhibitory signals (38). Recent discoveries found that CD244 can influence additional immune cells, such as dendritic cells (DCs). In particular, CD244 inhibits DCs, in that it inhibits the DC-induced inflammatory response and prevented DC-mediated activation of T cells and NK cells (39). Initial research did not consider the effect of CD244 on monocytes, but recent studies of patients with systemic lupus erythematosus (SLE) indicated decreased expression of CD244 in monocytes (see below) (40, 41). This review assesses the clinical significance of CD244 and its effects on the functions of various immune cells (Table 1) and in a wide range of diseases (Figure 2).

**Table 1:** The expression pattern of CD244/CD48 and related adaptor protein.

**Target cell**	**CD244 expression**	**CD48 expression**	**CD244 adaptor protein**
NK cell	+	+	SAP, ERT, EAT-2
CD4+ T cell	+	+	?
CD8+ T cell	+	+	SAP
Mast cell	+	+	?
Eosinophils	+	+	?
Monocyte	+	+	SAP, EAT-2
Macrophage	+	+	SAP, EAT-2
DC	+	+	SAP, EAT-2
Basophils	+	+	?

*Both of CD244 and CD48 are expressed in almost all the immune cells, and related adaptor proteins are shown*.

*+ means expressed in the corresponding cells (CD244 or CD48). ? means unclear*.

## CD244 in Autoimmune Diseases

### Systemic Lupus Erythematosus

SLE is an autoimmune disease of unknown etiology that primarily occurs in women (42). At the molecular level, SLE is characterized by the production of B-cell-dependent autoantibodies driven by CD4^+^ T cells and the formation of immune complexes that ultimately cause tissue damage (43). CD244 plays a negative regulatory role in humoral autoimmunity. Studies of *SLAMF4*^−/−^ mice indicated they had a higher ratio of activated lymphocytes and higher serum immunoglobulin levels than normal mice. Furthermore, this mouse model of SLE indicated the *SLAMF4*^−/−^ mice had more severe pathology. The subsequent immunodepletion was unrelated to the humoral immune regulation of CD244 and NK cells. In other words, although most functional studies of CD244 focused on NK cells, and NK cells can affect the functions of CD4^+^ T cells and B cells, CD244 acts independently of NK cells in the dysregulated humoral immunity that occurs during SLE (44).

However, the interaction of CD244 and CD8^+^ T cells plays two different roles in SLE patients. Kim et al. demonstrated that CD244 was highly expressed in IL-7Rα^low^ effector memory (EM) CD8^+^ T cells, which were more cytotoxic than IL-7Rα^high^ EM CD8^+^ T cells, and concluded this could reflect differences in SLE disease activity (45). Subsequent studies by Kis-Toth et al. (46) showed that SLE patients had a reduced number of SLAMF4^+^ CD8^+^ T cells, reduced SAP expression, and reduced cytotoxicity compared with healthy donors. Therefore, further research is needed to clarify differences in the expression of CD244 in different CD8^+^ T cell subsets. However, both of these studies showed that CD8^+^ T cells with higher expression of CD244 were more cytotoxic (46). Moreover, SLE patients have decreased expression of CD244 in monocytes and NK cells. Other research of CD244-deficient mice showed that regulation of NK cells by CD244 might be related to the gender-specific immune response in SLE (41, 47, 48). Genetic studies indicated that a single nucleotide polymorphism (SNP) in CD244 was related to SLE susceptibility, and that alternative splicing of the *CD244* mRNA also regulated SLE progression (49, 50). Further studies are needed to identify the roles of CD244 in the different cell types that function in the pathogenesis of SLE by use of mice with cell-specific CD244 gene knockout.

### Rheumatoid Arthritis

Rheumatoid arthritis (RA) is another inflammatory autoimmune disease that is also more common in females (50). A recent study showed that expression of various NK receptors, including CD244, by CD4^+^CD28^−^ T cells in RA patients enhanced the immune response, but this did not occur in healthy people (51). CD4^+^CD28^−^ T cells are highly differentiated EM CD4^+^ T cells. Autoantibody stimulation often leads to loss of CD28 and the production of cytokines and cytotoxic functions that differ from those of conventional CD4^+^ T cells (52). Research found that these cells aggravated inflammation and were present in the blood and synovial fluid of RA patients. The effect of CD244 on enhancing the function of these cells led to disease exacerbation and increased disease progression. Moreover, CD244 occurs in the monocytes of RA patients, and these monocytes can secrete TNF, which promotes the pathogenesis, whereas experimental blocking of CD244 reversed this phenotype (53). These results suggest that CD244 promotes the progression of RA. Genetic studies reported that a SNP of the CD244 gene correlated with RA susceptibility in Japanese and European Caucasian populations, but not in a Korean population (54–56). More studies are needed to clarify the function of CD244 in the initiation and progression of RA.

### Type 1 Diabetes

T1D is an autoimmune disease in which the immune system disrupts the normal function of pancreatic β cells (57). Researchers initially believed that the abnormal activation of DCs by pancreatic β cells led to an immune response of T cells, B cells, and macrophages. Subsequent researchers reported that NK cells participated in the autoimmune regulation of T1D by interacting with activated protein C (APC) (58, 59). Although there is no direct evidence for a role of CD244 in T1D, CD244 is likely to affect the immune regulation process of T1D by affecting the function of these immune cells. This hypothesis is based on observations of the expression of CD244 in various immune cells, and needs verification by subsequent studies.

The liver X receptor (LXR) is a nuclear receptor that affects various metabolic pathways *in vivo*. There is increasing evidence that an LXR agonist can be used to treat T1D (60, 61). Some studies found that the presence of CD244 in monocytes was an indicator of LXR activation, and that application of an LXR agonist increased the expression of CD244 (62). This indirect connection suggests the need for future studies of the specific functions of CD244 in T1D.

## CD244 in Infectious Diseases

### Viral Infections

Initial reports on X-linked lymphoproliferative disease type 1 (XLP1) indicated that CD244 was associated with antiviral immunity. The lack of SAP in XLP1 patients meant that CD244 in NK cells and T cells did not transmit an activation signal, and thus failed to kill B cells infected with the Epstein-Barr virus (EBV) (14, 63, 64). Subsequent studies found that the expression of CD244 in NK cells and virus-specific CD8^+^ T cells were altered during chronic viral infections from HIV, HBV, HCV, and HTLV-1. Thus, the expression level of CD244 and how it binds to the downstream proteins (SAP or EAT2) are critical factors for anti-virus immunity in different immune cell. And CD244 may function differentially in different virus infection (65).

Virus-specific CD8^+^ T cells are among the most effective defenses against viral infections because they kill infected cells by secreting cytokines or degranulation. Researchers found high expression of CD244, which has an inhibitory function, on CD8^+^ T cells. Furthermore, these cells had reduced SAP expression, so that CD244 transmitted stronger inhibitory signals to T cells, thus hindering the function of CD8^+^ T cells. Inhibition of CD244 enhanced the proliferation and cytokine secretion by CD8^+^ T cells (66–68). Raziorrouh et al. found that CD244 was co-expressed with PD-1, a common inhibitory receptor on the surfaces of T cells, and this enhanced the inhibition of HBV-specific CD8^+^ T cells in patients (69). In addition to inhibiting viral-specific CD8^+^ T cells, other research found over-expression of CD244 by CD4^+^ invariant natural killer T (iNKT) cells in HIV patients, with reduced secretion of IFN-γ, indicating the iNKT cells were also inhibited. Moreover, the expression of CD244 was significantly reduced after antiretroviral therapy, manifested by the decreased secretion of cytokines (IFN-γ, TNF-α, and others) (70).

Although CD244 expressed on the surfaces of virus-specific CD8^+^ T cells served as an inhibitory receptor in most chronic viral infections, it exhibited quite different regulatory function in HTLV-1 infections. HTLV-1 is a retrovirus, most infected individuals are asymptomatic carriers, and only a few develop adult T cell leukemia/lymphoma (ATL), a chronic progressive neurological disease termed HTLV-1-associated myelopathy/tropical spastic paraparesis (HAM/TSP) (71). CD244 had the different effects on virus-specific CD8^+^ T cells in ATL and HAM/TSP. Some research of HAM/TSP patients showed that the expression of CD244 and SAP in CD8^+^ T cells was significantly increased in HAM/TSP patients compared to healthy individuals, and that blocking CD244 with anti-CD244 antibodies inhibited degranulation and secretion of IFN-γ by CD8^+^ T cells (71). Subsequent research by Ezinne et al. identified an inhibitory effect of CD244 on CD8^+^ T cells (72). This difference may be ascribed to the different functions of CD8^+^ T cells in ATL and HAM/TSP. In patients with HAM/TSP, viral-specific T cells can kill virus-infected cells, but can also promote bystander activation and killing of nearby resident glial cells, and thereby aggravate the inflammatory response in the central nervous system.

In most cases of chronic viral infections, CD244 stimulates NK cells. During the early phase of HBV infection, expression of CD244 and SAP by NK cells decreases, resulting in impaired function of these cells. Anti-TGFβ1 treatment restores the expression of CD244 and the function of NK cells (73). A similar effect was reported in HIV infections. However, treatment for 12 to 36 months restored the expression of CD244 to a normal level, and this restored expression of CD244 was associated with recovery from NK cell cytotoxicity (74).

### Tuberculosis

Tuberculosis (TB) is responsible for more than 1 million deaths per year worldwide, and is the second leading cause of death from infectious diseases. However, most people infected with *Mycobacterium tuberculosis* (Mtb) are asymptomatic, and only 5 to 10% of those infected eventually develop active TB (75, 76). The course of TB infection depends on the host's immune response, and the immune function of CD244 affects the development of active TB. CD8^+^ T cells rely on IFN-γ and TNF-α to fight Mtb infections. Studies indicated that CD244 inhibited the expression of these two cytokines by up-regulating the expression of intracellular lncRNA-CD244 and recruiting the promoter of EZH2 to the INF-gamma and TNF-α genes (77). In contrast to antiviral immunity, CD4^+^ T cells play an important role in protective immunity against TB infections. Researchers found significantly increased expression of CD244 by the CD4^+^ T cells of patients with active TB rather than latent TB. However, production of IFN-γ by these CD4^+^ T cells was inhibited, suggesting upregulation of the inhibitory receptor of CD244 on CD4^+^ T cells during active TB (78). Another study reported that MDSC cells that expressed CD244 were more abundant in the peripheral blood of active TB patients, and this inhibited CD4^+^ and CD8^+^ T cells (15). These results indicate that CD244 plays an immunosuppressive role in active TB. Studies of animal models of TB are limited because of the unique infectivity and pathogenicity of TB in humans.

### Sepsis

Sepsis is the systemic organ dysfunction that occurs when an infection causes an extreme inflammatory response due to an over-active immune system (79), and is a major cause of death worldwide. Patients with immunosuppressed status have an increased risk for sepsis (80). Therefore, researchers have examined the effect of co-inhibitory molecules on the surfaces of immune cells as treatments for sepsis. Many studies have examined repression of the PD-1/PD-L1 immune checkpoint in sepsis, and there is evidence that excessive expression of these proteins can lead to T cell dysfunction or failure. Moreover, drugs that block PD-1/PD-L1 can provide effective treatment of sepsis. CD244 plays a similar role in sepsis, as a co-inhibitory molecule that interacts with PD-1 (16). Chen et al. found high expression of CD244 on the CD4^+^ T cells of patients with sepsis, and that this inhibited the function of these cells, reduced the activation of macrophages, and provided an immunosuppressive effect (81). Thus, like most co-inhibitory molecules, CD244 plays a crucial role in establishment of the immunosuppressive environment in patients with sepsis. Small molecule or siRNA that could inhibit the expression or block the functional activity of CD244, either alone or in combination, may therefore be an effective therapeutic strategy for sepsis (81, 82).

## CD244 in Allergy

The prevalence of allergic diseases is increasing worldwide with the changing lifestyle, environmental factors, and increasingly diverse food, especially the immediate allergic reaction (83). Immediate allergic reactions are mainly caused by IgE mediated degranulation of mast cells. The granules released by mast cells contain histamine, heparin and other bioactive substances. Excessive degranulation will contribute to the body's capillaries dilate and enhance vascular permeability, local edema, and thus the other symptoms of inflammation (84, 85). Eosinophils are another important immune cell involved in allergic reaction. They can be recruited to the inflammatory site under the action of inflammatory mediators and chemokines, and release large amounts of prostaglandin E, leading to tissue damage and inflammation (86). The physical cross-talk between mast cells and eosinophils is critical for the development of allergic diseases (87, 88). Although the roles mast cells and eosinophils play in the progress of allergic diseases have been studied for many years and achieved much progress, further studies are needed to fully clarify the mechanisms and develop novel therapies in the clinical.

CD244 is expressed in eosinophils and CD48 in mast cells. An *in vitro* study suggested that the expression of CD244 was required for eosinophil adhesion and chemotaxis, and its surface expression was upregulated in allergic rhinitis (AR) after challenge. Moreover, AR patients had increased expression of CD244 (89). However, more studies of allergic diseases concentrated on CD48 rather than CD244. The interaction between CD244 and CD48 is the basis for the coaction of these two cell types. The activation of eosinophils depends on the interaction of CD48 (on mast cells) and CD244 (on eosinophils), and enhancing the function of either protein can activate mast cells and the interactions between the two cell types (4, 90). This leads to the conclusion that CD244 is critical for the initiation and continuation of allergic status, and that blockade of either CD244 or CD48 is a promising therapeutic strategy for treatment of allergic diseases.

## CD244 in Tumors

### Tumor Related Immune Cells

The function of immune cells in the microenvironment of the tumor significantly influences tumor outcome. There is evidence that CD244 is primarily expressed by NK cells, T cells, MDSCs, and DCs in the tumor microenvironment and that it functions in the regulation of these cells (48, 91–93). By recognizing MHC-I on the surfaces of normal cells, mature NK cells can kill tumor cells without damaging normal cells, whose function is regulated by multiple inhibitory and stimulatory signals (94). Research indicated that CD244 had opposing regulatory effects on NK cells in different cancers. In particular, a study reported that CD244 was downregulated in the NK cells of patients with acute myeloid leukemia (AML), and that NK cell activity positively correlated with progression-free survival of these patients (95). Hoffmann et al. found that CD244 mediated the adhesion of NK cells to HeLa cells, and was responsible for the cytotoxic effect of NK cells on tumor cells (96). However, studies of patients with hepatocellular carcinoma reported that NK cells developed exhaustion following their interaction of CD244 (on NK cells) and CD48 (on monocytes) (97). CD244 can also prevent NK cells from achieving anti-tumor immunity by regulating the function of DCs. Animal studies showed that NK cells were more active in CD244^−/−^ mice than wild-type (WT) mice (39, 98). These studies provide evidence NK cells are affected by different types of other immune cells in distinct tumor microenvironments. This may partially explain their dual roles in anti-tumor immunity.

T cells have an essential role in tumor immunity, and CD244 is expressed by CD4^+^ and CD8^+^ T cells, thereby regulating the anti-tumor effect of these cells or mediating immune escape in mice (99). The expression of CD244 in CD4^+^ and CD8^+^ T cells was increased in a mouse model of lung cancer, and these cells exhibited more apoptosis and decreased secretion of anti-tumor cytokines. This indicated that CD244 functioned as an inhibitory receptor in lung cancer (100). A study of chronic lymphocytic leukemia (CLL) patients reported that CD244 was highly expressed and acted as a marker of T cell exhaustion, in that it was associated with a significant reduction in T cell cytotoxicity and proliferation; however, the production of cytokines (IFN-γ and TNF-α) remained unchanged, so these researchers considered this a “pseudo-failure” of T cells due to continuous stimulation of low-affinity autoantigens (101). The effect of “pseudo-failure” of T cells with elevated CD244 expression on tumor outcome needs further examination. There is also evidence that CD244 does not directly stimulate the proliferation and activation of tumor-specific T cells, but acts as a co-stimulus that enhances the antigen activation reaction of tumor-specific T cells by enhancing the T cell receptor signal (102).

MDSCs have a strong immunosuppressive function, and also play an inhibitory role in tumor immunity. Isolation of MDSCs from tumor-bearing mice showed differences in functional activity between CD244^+^ MDSCs and CD244^−^ MDSCs, in that the former cells had significant inhibitory activity against CD8^+^ T cells (93, 103).

### Tumor Immunotherapy

The field of tumor immunotherapy is developing rapidly and includes several approaches, such as checkpoint inhibition, cancer vaccines, and adoptive immunotherapy (104). CD244 is a regulatory receptor present in various immune cells, and can therefore be regarded as an immune checkpoint that regulates the immune response and kills tumor cells. CD244 can also be used for construction of a chimeric antigen receptor (CAR) on NK cells. CAR-NK immunotherapy is a novel adoptive immunotherapy that was developed following CAR-T cell immunotherapy. Like CAR-T cells, CAR-NK cells also target tumors and have enhanced anti-tumor function (105, 106). Although there are FDA-approved CAR-T cell therapies, these treatments have some limitations. The major side effects are killing of healthy autologous T cells and tumor recurrence caused by antigen loss. In contrast, CAR-NK cells do not kill autologous T cells and can still be activated when the tumor antigens are lost. The construction of a co-stimulation domain can also increase the tumor-killing activity of CAR-NK cells. Therefore, CAR-NK cells appear to have greater potential for use in adoptive immunotherapy (106–108). The introduction of CD244 as a critical co-stimulant molecule on the surfaces of NK cells, as a second and third generation CAR structure, can significantly enhance the anti-tumor effect of CAR-NK cells and reduce the inactivation of CAR-engineered cells caused by some tumor cells (109).

## Conclusion and Prospect

CD244 is a member of the SLAM family that transmits different cell transduction signals through different pathways on various immune cells, and thereby regulates the functions of these immune cells and affects immune responses in various diseases. Previous studies indicated that CD244 can affect the onset and progression of autoimmune diseases (SLE and RA), chronic viral infections, tuberculosis, sepsis, and other diseases by altering the function of NK cells, T cells, monocytes, DCs, MDSCs, and other immune cells. An important feature of CD244 is that it can transmit different signals through different intracellular transduction pathways, so that it has different regulatory effects on immune cells and can have opposing effects on the development of different diseases. In the tumor microenvironment, these changes in immune cell function can directly affect the fate of tumor cells. There is also evidence that using CD244 as the co-stimulation domain to construct CAR-NK cells for adoptive immunotherapy could significantly improve the anti-tumor activity of CAR-engineered cells.

In addition to the diseases described above, CD244 also appears to be associated with several other diseases. For example, recent studies suggested that the balance of intestinal flora affects many immune-related diseases, including cancers. Some recent studies showed that intestinal flora controls the function of immune cells by inducing the expression of CD244 by immune cells present in the intestinal mucosa, and that this maintains the biodiversity of intestinal flora and affects host immune function (110). Thus, therapeutic alteration of the expression of CD244 may potentially affect the intestinal flora and restore the normal balance. Allergic diseases such as allergic rhinitis (AR) are primarily characterized by increased levels of eosinophils, and treatment of these diseases remains challenging. Recent studies found that the expression of CD244 by eosinophils induced adhesion and chemotaxis, and stimulated eosinophils (89). Therefore, therapeutic alteration of the expression of CD244 may change the characteristics and functions of eosinophils, and potentially provide a new treatment for AR and similar diseases (111).

However, research has also indicated a number of unresolved problems regarding CD244. For example, studies of SLE patients showed that CD244 had different regulatory effects in the same type of immune cells (CD8^+^ T cells) (46, 47); some studies attributed this difference to the different regulatory effects of CD244 by different cell subtypes. However, this problem is not so simply resolved because CD244 also has different regulatory effects on virus-specific T cells in two diseases caused by HTLC-1 (ATL and HAM/TSP), and had adverse effects in both cases. This phenomenon could be explained by the different roles of virus-specific T cells in these two diseases, but more detailed studies of the regulatory mechanisms are necessary.

In any case, the present summary of existing research indicates that CD244 functions as either a stimulatory or an inhibitory molecule that is common on immune cells, and plays an important regulatory role in the onset and progression of various immune-related diseases. Ascertainment of the detailed mechanism of CD244 in different diseases, its use as a prognostic marker, and its use in precise immunotherapy regimens that target CD244 are important directions for future research.

## Author Contributions

GW developed the main concepts of the manuscript. LS and XG wrote the first draft. XZ and ZL contributed to editing. TZ and SZ contributed to revision of final draft. All authors read and approved the submitted version.

## Conflict of Interest

The authors declare that the research was conducted in the absence of any commercial or financial relationships that could be construed as a potential conflict of interest.
